# Expression, immunolocalization and processing of fertilins ADAM-1 and ADAM-2 in the boar (*sus domesticus*) spermatozoa during epididymal maturation

**DOI:** 10.1186/1477-7827-9-96

**Published:** 2011-06-30

**Authors:** Anna Fàbrega, Benoît Guyonnet, Jean-Louis Dacheux, Jean-Luc Gatti, Marta Puigmulé, Sergi Bonet, Elisabeth Pinart

**Affiliations:** 1 Biotechnology of Animal and Human Reproduction (TechnoSperm), Department of Biology, Institute of Food and Agricultural Technology, University of Girona, Campus Montilivi, s/n, 17071 Girona, Spain; 2 Gamètes Males et Fertilité, Physiologie de la Reproduction et des Comportements, UMR 6175 INRA-CNRS-Université de Tours, 37380 Nouzilly, France; 3 ESIM, UMR 1301 IBSV INRA-CNRS-Université Nice Sophia Antipolis, 400 route des Chappes, 06903 Sophia Antipolis, France

## Abstract

Fertilin alpha (ADAM-1) and beta (ADAM-2) are integral membrane proteins of the ADAM family that form a fertilin complex involved in key steps of the sperm-oocyte membrane interaction. In the present work, we analyzed the presence of ADAM-1 and ADAM-2 mRNAs, the spermatozoa proteins' processing and their sub-cellular localization in epididymal samples from adult boars. ADAM-1 and ADAM-2 mRNAs were highly produced in the testis, but also in the *vas efferens *and the epididymis. On immunoblots of sperm extracts, ADAM-1 subunit appeared as a main reactive band of ~50-55 kDa corresponding to occurrence of different isoforms throughout the epididymal duct, especially in the corpus region where isoforms ranged from acidic to basic pI. In contrast, ADAM-2 was detected as several bands of ~90 kDa, ~75 kDa, ~50-55 kDa and ~40 kDa. The intensity of high molecular mass bands decreased progressively in the distal corpus where lower bands were also transiently observed, and only the ~40 kDa was observed in the cauda. The presence of bands of different molecular weights likely results from a proteolytic processing occurring mainly in the testis for ADAM-1, and also throughout the caput epididymis for ADAM-2. Immunolocalization showed that fertilin migrates from the acrosomal region to the acrosomal ridge during the sperm transit from the distal corpus to the proximal cauda. This migration is accompanied by an important change in the extractability of a part of ADAM-1 from the sperm membrane. This suggests that the fertilin surface migration may be triggered by the biochemical changes induced by the epididymal post-translational processing of both ADAM1 and ADAM-2. Different patterns of fertilin immunolocalization then define several populations of spermatozoa in the cauda epididymis. Characterization of such fertilin complex maturation patterns is an important step to develop fertility markers based on epididymal maturation of surface membrane proteins in domestic mammals.

## Background

Modern breeding programs use artificial insemination with a low number of males for improving the livestock genetics of economically important traits. However, some of the interesting males have a low fertility even when classical semen parameters (i.e. viability, motility, abnormal forms...) are normal. It is thus important to develop new molecular tools to accurately estimate fertility levels. As the quality of the epididymal maturation strongly influences sperm fertility, sperm protein markers of this maturation are among the most promising tools. However, only few sperm surface maturation proteins with a role in fertility have been described in large mammals since almost all studies were performed on rodents' models. Among the spermatozoa surface proteins, fertilin, an heterodimer complex composed of two integral membrane glycoproteins named alpha-fertilin (ADAM-1) and beta-fertilin (ADAM-2) (also previously named PH-30 alpha and PH-30 beta in guinea pig), as well as several other ADAMs have been reported to be involved in sperm-oocyte recognition and in membrane fusion [1-5]. Both proteins are members of the "A Disintegrin And Metalloprotease" domain protein family (ADAMs) [1] whose sequences contain a pro-domain, a metalloprotease, a disintegrin and a cysteine-rich domain, EGF-like repeats, a transmembrane domain and a carboxy-terminal cytosolic tail. The fertility of male mice lacking fertilin alpha or fertilin beta is substantially reduced due to sperm inability to migrate through the oviduct and to bind to the zona pellucida and to the oocyte plasma membrane [4-8]. It has been suggested that the binding between the disintegrin domain of ADAM-2 and the egg plasma membrane integrin(s) is at least partly responsible for the recognition between sperm and eggs.

In rodents, the fertilin complex can be found in testicular germ cells under the form of a 160-190 kDa precursor composed of pro-alpha and pro-beta subunits assembled into a non-covalently bound complex [9]. The pro-alpha subunit is proteolytically processed into a 50 kDa protein by a pro-protein convertase during spermiogenesis, before emerging on the sperm cell surface. Its molecular mass does not change further during epididymal sperm maturation [2,9]. In contrast, the pro-beta subunit is present as a full length protein on the testicular sperm surface and then proteolytically transformed during sperm maturation in the epididymis [2,9]. The pro-beta subunit maturation is characterized by the formation of intermediate forms during the passage of spermatozoa through the caput, that are cleaved into a 35 kDa main form in spermatozoa from proximal and distal cauda [9]. This proteolytic processing results in the removal of the pro- and metallo-protease-like domains, with only the full or part of the disintegrin domain, the cysteine-rich domain, the EGF repeat, the transmembrane and the cytoplasmic domains remaining on the sperm cell. This processing also induces a relocation of the fertilin complex on a different plasma membrane domain of the mature spermatozoa.

Most of the previous studies have been performed with guinea pig and mouse and although a similar pattern of fertilin modifications was suggested in the bull [10], the processing of the fertilin complex during the epididymal maturation in large domestic mammals has never been described in details. The aim of the present work was to characterize the fertilin complex processing throughout the epididymal duct of the boar, an economically important, yet fewly studied, farm animal. This study is a pre-requisite to address the question of the fertilin role in boar fertility and its potential use as a fertility marker in boars.

## Methods

### Reagents and chemicals

Bovine Serum Albumin (BSA), Nonidet P-40 (NP-40), proteases inhibitor cocktail (consisting of AEBSF, aprotinin, bestatin hydrochloride, E-64, EDTA, leupeptin hemisulfate salt (S8820, Sigma, L'Isle d'Abeau, France)) supplemented by 1 mM phenyl-methane-sulphonyl-fluoride (PMSF) just before use), Percoll, and common laboratory salts and reagents were purchased from Sigma. Ampholytes pH 2-11 (Servalytes) were obtained from Serva (Heidelberg, Germany) and ampholytes pH 3-10 (Ampholytes), Coomassie Brilliant Blue (Phastgel Blue R), and electrophoresis calibration kits (standard proteins) were purchased from GE-Healthcare (Saclay, France).

### Epididymal and testicular samples

Ten adult boars (Large White) aged from 8 months to more than 2 years were used throughout the study, following the approved guidelines for the ethical treatment of animals. Testis and epididymides were immediately removed after slaughter of the animals and dissected avoiding blood contamination (less than 15 minutes after death). For RNA extraction, samples were immediately frozen in liquid nitrogen and stored at -80°C until use. For polyclonal antibodies testing, testis tissues (about 100 mg) were grinded in 2 ml Phosphate Buffer Saline (PBS; 50 mM potassium phosphate; 150 mM NaCl; pH 7.2) containing 2% Triton X100 and proteases inhibitor cocktail, and then centrifuged 10 min at 15 000 g. An aliquot of the supernatant was mixed with reducing Laemmli 4X sample buffer, boiled, and stored at -20°C until use.

**Figure 1:**
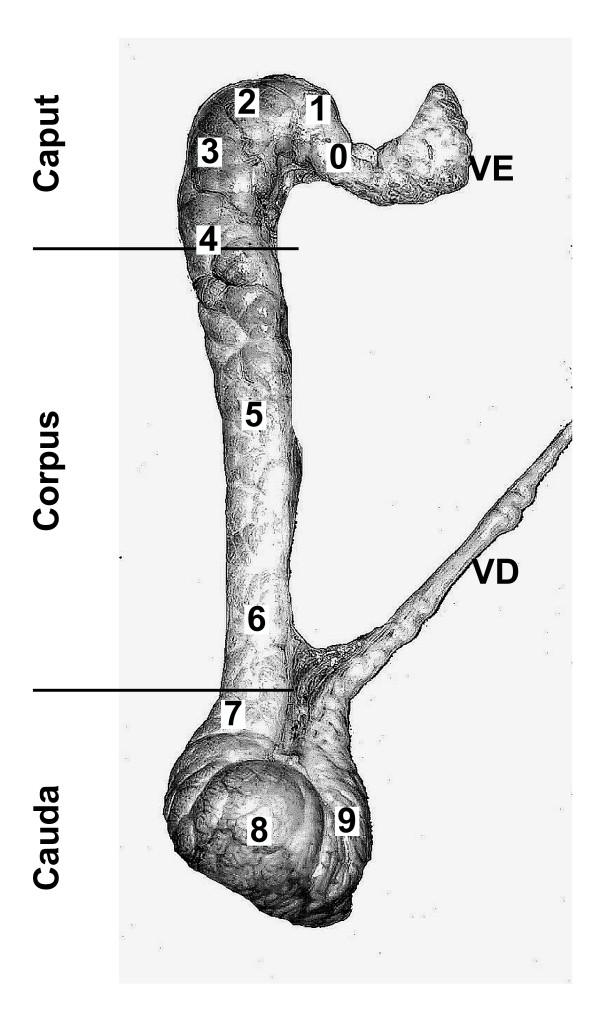
**Adult boar epididymis**. Picture of boar epididymis indicating the different regions used: VE, *vas efferens*; 0 to 8/9, epididymal zones; VD, *vas deferens*, and the three main classical regions: caput, corpus, cauda.

Testicular fluid and spermatozoa were obtained by puncture from the rete testis. Epididymal spermatozoa and fluids from the different boar epididymal regions (Figure 1) were obtained through microperfusion with supplemented phosphate buffer saline (PBS supplemented with 2 mM pyruvate, 2 mM lactate, 2 mM glucose and 5 mM K_2_CO_3_) as described in previous studies [11-15]. Fluid and spermatozoa from *vas deferens *and region 9 were obtained by retroperfusion from the *vas deferens *with supplemented PBS. These techniques allow obtaining samples without blood and extracellular fluid contaminations [15].

Samples were then centrifuged at 600 × g for 10 min at 15-20°C. Sperm pellets were resuspended and washed at 600 × g for 10 min at 15-20°C with supplemented PBS and, when necessary, separated on a 40-90% Percoll gradient (Sigma) to obtain very clean sperm samples. Then, sperm viability was assessed using SYBR-14 and propidium iodure (PI) (LIVE/DEAD^®^Sperm Viability kit, Molecular Probes, Invitrogen, Illkirch, France) in flow cytometry experiments (Cell Lab QuantaSC, Beckman Coulter, Villepinte, France). This ensured that all samples had retained sperm vitality over 80%. For some experiments, sperm samples were divided into two aliquots for total protein extraction and for protein surface labeling, respectively.

### Total spermatozoa protein extraction

Total protein extraction was performed by mixing sperm suspension (in average 10^8 ^to 10^9 ^spermatozoa) with PBS-2% SDS containing a proteases inhibitor cocktail (v/v), or directly by mixing the washed sperm with an equal volume of Laemmli 4X buffer without β-mercaptoethanol. The samples were incubated with the extraction buffer for 30 min at 4°C under agitation, and then centrifuged at 15 000 × g for 15 min at 4°C. The supernatant was carefully removed and stored for a short period of time at -20°C.

### Protein surface labeling with biotin and purification

Labeling was done mainly as described in Belleannèe et al. (2011) [16]. Sperm samples were resuspended in supplemented PBS at a final concentration of 3 × 10^9 ^spz/ml. Labeling was performed with 3 mg/ml (~ 6 mM) of NH-SS-Biotin (Thermo Scientific, Pierce, Brebières, France) during 30 min at room temperature under agitation. Biotin labeling was stopped with 50 mM Tris or 300 mM glycine. Biotin excess was eliminated by successive washes with supplemented PBS followed by a 40-90% Percoll gradient. Proteins were extracted by mixing the sperm pellet with an equal volume of PBS with 2% (v/v) Nonidet P-40, a proteases inhibitor cocktail, and 1 mM PMSF during 30 min at 4°C under agitation. Then, the samples were centrifuged at 15 000 × g for 15 min at 4°C and the supernatant was carefully removed and stored at -20°C. The biotin-labeled surface proteins were purified using streptavidin beads (Dynabeads^®^M-280 Streptavidin, Invitrogen) and extracted from beads by boiling for 5 min in Laemmli buffer.

### Gel electrophoresis

For SDS-PAGE separations using 8-16% gradient gels, samples were diluted v/v in Laemmli buffer 4X with 5% β-mercaptoethanol and boiled during 3 min at 95°C. A preliminary gel was run with the extracts and stained with Coomassie blue in order to equilibrate protein amounts for the western blot experiments (not shown).

For isoelectrofocalisation, immobiline strips (7 cm long Ready Strip IPG pH 3-10, Biorad) were first rehydrated overnight at 20°C in presence of the protein extract mixed with the following buffer: urea 7 M, thiourea 2 M, Triton X-100 1%, N-octyl-β-D-glucopyranoside 2%, DTT 30 mM, ampholytes (IPG Buffer pH 3-10, Amersham) 0.4%, and bromophenol blue. For isoelectrofocalization the following program was applied: 100 V 10 min; lineal gradient of 100 V to 4000 V during 7 h 30 min; constant voltage of 4000 V during 9 h; fast gradient from 4000 V to 8000 V during 1 h 30 min; final step at 500 V.

Before SDS-PAGE electrophoresis, the strips were rinsed with two equilibration solutions, equilibration solution I (urea 6 M, Tris 50 mM, glycerol 30%, SDS 2%, DTT 65 mM) and then equilibration solution II (urea 6 M, Tris 50 mM, glycerol 30%, SDS 2%, DTT 135 mM), during 15 min each, at room temperature under agitation, in order to reduce and to alkylate the proteins. The strips were deposited on the top of a 8-16% gradient gel and casted with a 0.5% agarose gel dissolved in electrophoresis buffer with bromophenol blue. The electrophoresis was run at 30 mA per gel.

### Antibodies

Four different rabbit polyclonal antibodies against ADAM-1 and ADAM-2 were prepared from defined peptides. Two peptides, one in the N-terminal metalloprotease domain and one in the C-terminal part near the trans-membrane domain were chosen for each ADAM protein (ADAM-1-NT: LTEVPVDLQVALRC; ADAM-1-CT: CSSPGSGGSVDSGP; ADAM-2-NT: GLTNAIFTSFNITC; ADAM2-CT: NATITYSNINGKIC). A cysteine was added in C-terminal position of the peptides that did not have one for the coupling with keyhole limplet hemocyanin (Sigma) using a bi-functional coupling agent (MBS, Pierce). 250 μg of coupled peptide were used for each injection into rabbit, using Freund complete adjuvant for the first injection and Freund incomplete adjuvant for the three next injections. The preimmune serums were tested prior immunization and showed no reactivity toward the testicular and sperm extracts (not shown). Antibody specificity was also demonstrated by competition assays between the peptide and the antibody on Western blots from testis extract (see example in Additional file 1, Figure S1). The rabbit polyclonal anti-germinal angiotensin-converting enzyme (anti-ACE) has been described previously [17]. Immunized rabbit sera were used throughout the study.

### Protein blotting

After one or two-dimensional electrophoresis, gels were transferred to nitrocellulose or polyvinylidene fluoride membranes (Immobilon-P, Millipore, Molsheim, France) during 2 hours at 0.8 mA per cm^2^, stained with Ponceau red to check for transfer homogeneity, and then blocked during 1 hour at room temperature with TBS-Tween (Tris HCl 20 mM, NaCl 150 mM, 0.5% Tween 20, pH 7.3) containing 5% low fat milk.

Blocked membranes were incubated with the first antibody diluted 1/5000 (anti-ADAMs) or 1/10000 (anti-ACE) in blocking solution overnight at 4°C, and then, incubated after washing with a goat anti-rabbit-HRP (Sigma) diluted 1/5000 in blocking solution during 1 h at 37°C.

Reactive bands were visualized either with a chemiluminescent substrate (Immobilon™ Western Detection Reagents, Millipore) with exposition of membranes with photographic films (Hyperfilm, GE Healthcare), or directly digitized with an image analysis device or photographed after 4-chloro-1-naphtol staining.

### PCR analysis

For PCR, tissues treatment and reverse transcription were performed as described in Guyonnet et al, (2009) [18]. 3 μg total RNA were retro-transcribed using the Superscript Reverse transcriptase H (Invitrogen) and oligo(dT)15 primers. PCRs were performed for 25 and 30 cycles at the temperature specified for the primer sets (ADAM-1 forward GGCCACATTAATGGCAGACT, ADAM-1 reverse ACATGCTTGGCAGGAAAATC; ADAM-2 forward GTGGGAATGGAGAAGTCGAA, ADAM-2 reverse GACATGCTGCAGAAGAACCA). Aliquots of each reaction mixture were analyzed on a 2% ethidium bromide-stained agarose gel. Amplification of RPL19 (Ribosomal protein L19) was used as PCR control (RPL19 forward GGTACTGCCAATGCTCGAAT, RPL19 reverse CCATGAGAATCCGCTTGTTT). Testicular ADAM-1 and ADAM-2 amplicons were sequenced in order to validate the primers (see Additional file 2, Figure S2).

### Immunocytochemistry

Boar sperm samples from proximal and distal caput, corpus and distal cauda were washed and fixed with 3% para-formaldehyde for 30 min at room temperature. After three washes in PBS at 600 × g for 10 min, samples were diluted ~10^7 ^spz/ml in PBS and dropped onto poly-lysine-coated slides, rinsed with PBS, and incubated for 1 h with the anti-C-terminal ADAM-1 polyclonal antibody diluted 1/100 in PBS-1% BSA. After washing in PBS, samples were incubated for 1 h with the secondary antibody goat anti-rabbit conjugated with Alexa Fluor 488 (Molecular Probes, Eugene, OR, USA), diluted 1/1000 in PBS-1% BSA. After extensive washings with PBS, the mounting medium (Fluoromount; Electron Microscopy Sciences, Hatfield, PA, USA) was applied. Digital images were captured at ×630 and ×1000 magnification under a fluorescence microscope with AxioVs40 v4.6.3.0 software (Zeiss Axio Imager.Z1).

Autofluorescence controls were spermatozoa incubated with PBS-1% BSA without antibodies, with primary antibody only (not incubated with secondary antibody) or with secondary antibody only (not incubated with primary antibody). No labeling was observed when the preimmune serum was used instead of the polyclonal antibody (see Additional file 3, Figure S3). The different patterns of fertilin localization were obtained by analyzing under the microscope 3 replicates of 100 spermatozoa per boar. Results were statistically analyzed with SigmaStat 3.5 statistical software. A one-way analysis of variance with post-hoc multiple comparison procedure (Holm-Sidak method) was performed.

## Results

### Pig ADAM-1 and ADAM-2 sequence and expression

**Figure 2:**
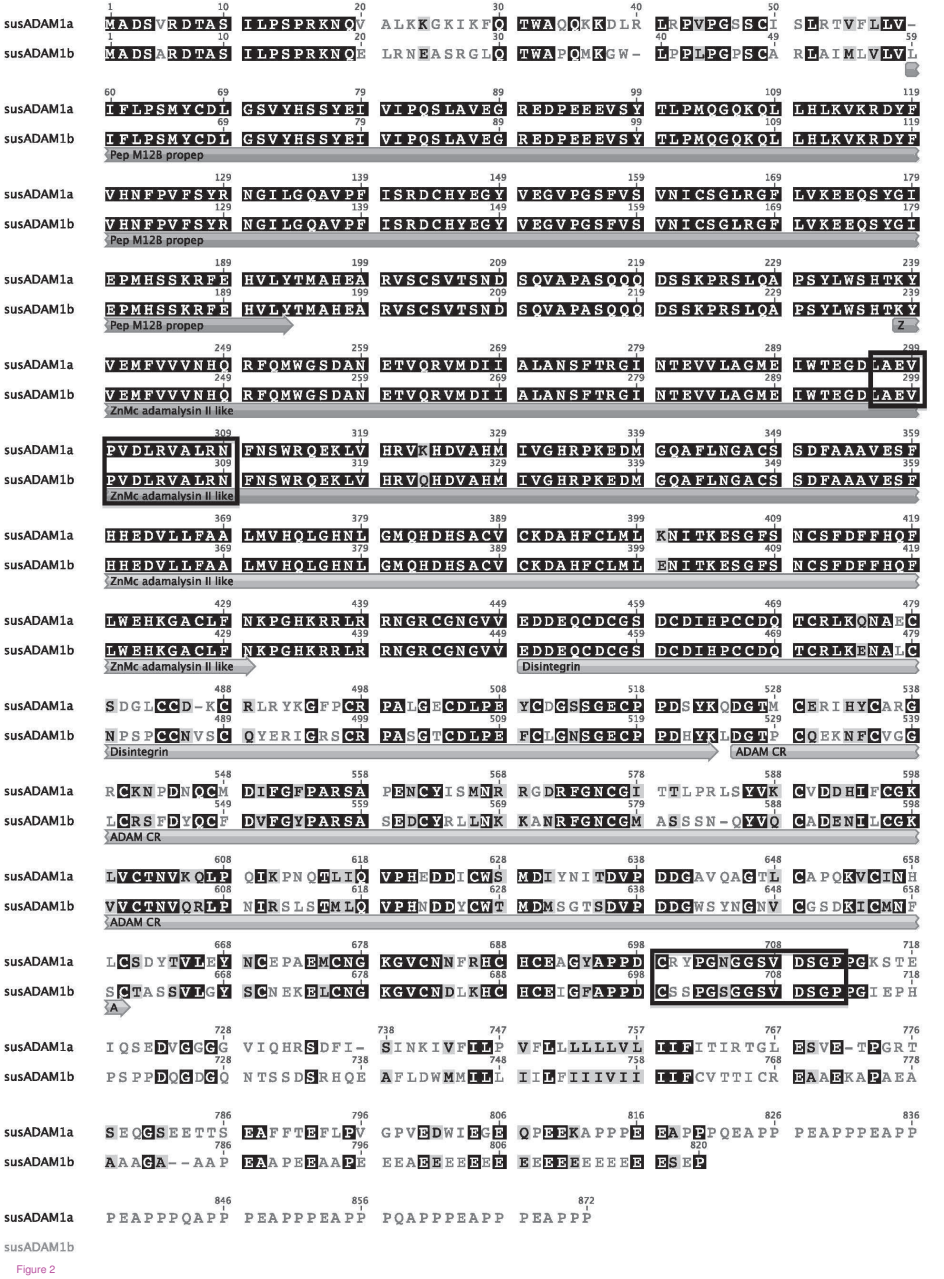
**Sequence comparison of the two pig ADAM-1**. Sequence alignment between pig ADAM-1a and ADAM-1b showing similarities (black boxes), homologies (grey boxes) and differences in amino acids. The position of the different ADAM domains is indicated. Positions of the N-terminal and C-terminal peptides used for immunization are indicated by boxes.

**Figure 3:**
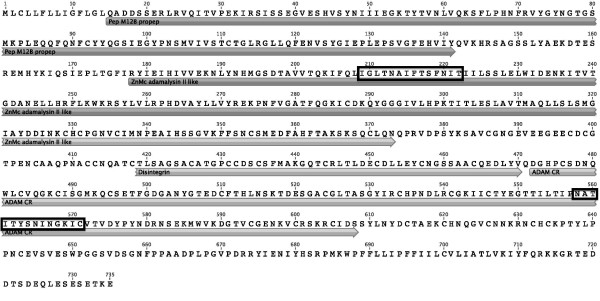
**Sequence of the pig ADAM-2**. Sequence of the pig ADAM-2 showing the position of the different ADAM domains. Positions of the N-terminal and C-terminal peptides used for immunization are indicated by boxes.

In order to design peptides for immunization, we searched databases for complete sequences of pig ADAM-1 and ADAM-2 (Figure 2 and 3). At that time, the complete sequence of pig ADAM-2 was available [19], but only a partial C-terminal sequence of ADAM-1 was found in databases. We then used these sequences to design specific N- and C-terminal peptides for ADAM-2 and C-terminal ADAM-1 peptide. The N-terminal ADAM-1 peptide sequence was designed in a highly conserved region using the alignment between the bull ADAM-1 sequence and rodents' sequences. Except for this peptide, other peptides used for immunization were then specific for the pig ADAMs and did not match any other protein in databases. Since then, an incomplete sequence of the pig ADAM-1 (XP_001924862.1) and an alternative ADAM-1 sequence (XP_001927304.1), named therein ADAM-1a and ADAM-1b respectively, were deposited on the public data bank (Figure 2). We have completed the sequence of the pig ADAM-1a by assembly with the best matching pig ESTs sequences available in public databases (*Sus scrofa *cDNA clone PDUts1113E12 and PDUts2081H12).

The pig ADAMs sequences showed the classical architecture of ADAMs family in terms of the presence of the different domains. Both ADAM-1 proteins have a predicted metalloprotease active site and a cleavage site for pro-protein convertase (436-KRRLR-440), which is not present on the ADAM-2 chain. At the protein sequence level, pig ADAM-1a shared only 74% identity with pig ADAM-1b, the main differences being found in the pro-domain, the disintegrin, the cysteine rich and the C terminal domains (Figure 2). Pig ADAM-1a was 71%, 69%, 64%, and 60% identical to horse ADAM-1 (XM_001490830.2), bull ADAM-1 (XM_866254.3), mouse ADAM-1a (NM_172126.2) and guinea pig PH-30 alpha (NM_001173096.1), respectively. Pig ADAM-1b had 68% identity with bull, 66% with horse, 59% with mice alpha-1a and alpha-1b, and 58% with guinea pig PH30 alpha, respectively. Pig ADAM-2 showed 65%, 65%, 57%, and 53% identity with horse (XM_001490821.2), bull (NM_174228.1), mouse (NM_009618.2) and guinea pig ADAM-2 (NM_001172910.1), respectively.

**Figure 4:**
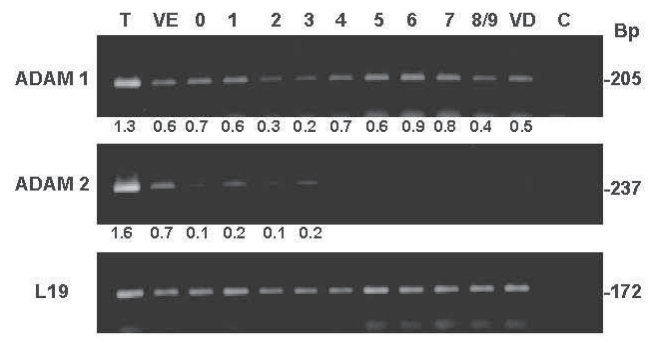
**Expression of ADAM-1 and ADAM-2 in testis and epididymis**. Agarose gel showing the PCR amplicons obtained for ADAM-1 and ADAM-2 using total RNAs extracted from the testis, *vas efferens*, *vas deferens *and the different epididymal zones, and 30 amplification cycles. The RPL19 gene was used as a control. The intensity ratio between ADAMs and RPL19 amplicons is indicated under each lane.

Primers were designed from pig ADAM-1 and ADAM-2 sequences in order to analyze by PCR the presence and expression level of their mRNAs within testicular and epididymal tissues from different regions (Figure 4). After 25 RT-PCR cycles, we observed the presence of both mRNAs only in the testis. After 30 cycles, ADAM-1 mRNAs were also detected at a slightly lower intensity in the *vas deferens *and throughout the epididymis while ADAM-2 mRNA was detected in the efferent ducts and at very low intensity from the caput to the corpus epididymis.

### ADAM-1 and ADAM-2 processing in testicular and epididymal spermatozoa

**Figure 5:**
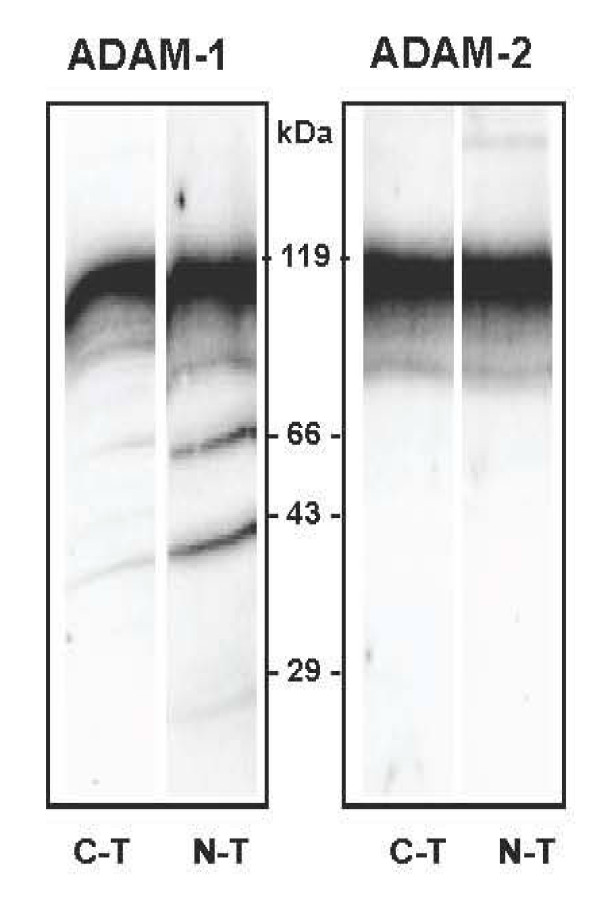
**Reactivity of the different anti-ADAMs polyclonal antibodies**. Boar testicular tissue extract was separated on a 8-16% SDS PAGE under denaturing conditions, and transferred to nitrocellulose. Individual lanes of the blot were probed with the four different anti-peptide polyclonal antibodies (ADAM-1 and ADAM-2 C-terminal (C-T) and N-terminal (N-T)).

The four anti-peptide polyclonal antibodies were first tested for specificity on total testis extracts (Figure 5 and Additional file 2, Figure S2). All antibodies reacted with main bands at about 100 kDa, slightly higher than the theoretical masses expected for the pig ADAM-1 (unprocessed expected mass 95 kDa) and ADAM-2 (unprocessed expected mass 82 kDa). This suggested occurrence of post-translational modifications, in agreement with the fact that glycosylation sites are predicted all over the sequence of both ADAM-1 and ADAM-2.

**Figure 6:**
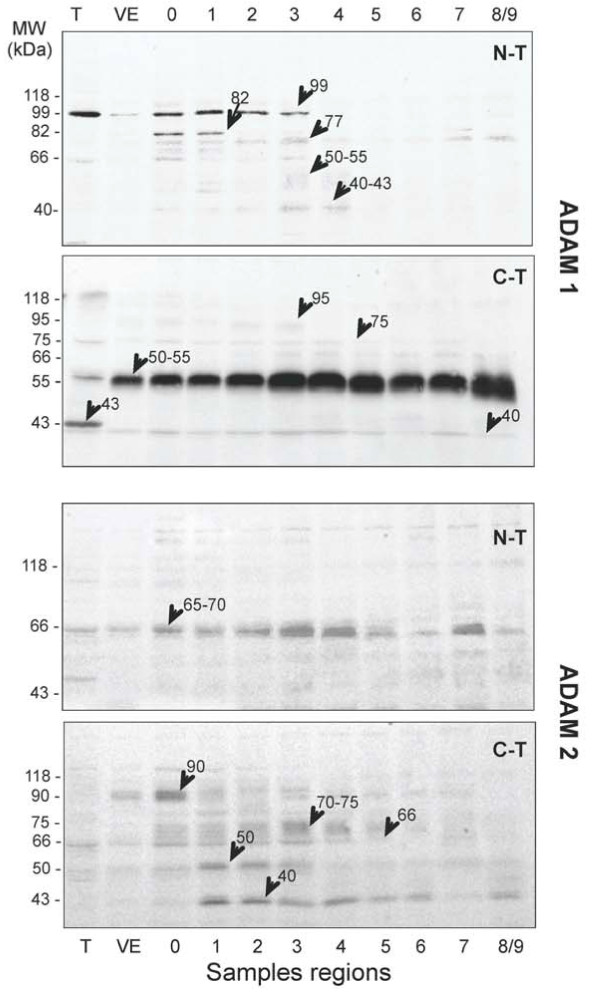
**Epididymal processing of ADAM-1 and ADAM-2**. Western blots loaded with equivalent total sperm extracts from the different epididymal zones were probed either with the ADAM-1 or ADAM-2 anti-N-terminal or anti-C-terminal polyclonal antibodies as indicated. Arrows and Mr on the figure indicate the main reactive bands (see results). Proteins amounts from about 10^6 ^sperm were loaded/lane.

In sperm extracts from the different epididymal regions (Figure 6), the anti-ADAM-1 N-terminal polyclonal antibody recognized one main band at 99 kDa from testicular to the distal caput (zone 3) spermatozoa extracts. Other bands, including a strong 82 kDa band could be transiently visible in zones 0 and 1, as well as several very faint bands such as a 77 kDa band until the cauda, or 50-55 and 40-43 kDa bands in zone 3-4.

The anti-ADAM-1 C-terminal polyclonal antibody reacts with five bands in testicular sperm with masses at about 118 kDa, 75 kDa, 50-55 kDa, 43 kDa and a faint 40 kDa. For epididymal sperm extracts, the 50-55 kDa band was the most reactive and it increased in intensity and spread largely in the cauda epididymal sperm extracts. Faint 95 kDa and 75 kDa bands that were barely visible in testicular sperm extracts almost disappeared in distal caput and cauda sperm, respectively. The 43 kDa band, present in testicular sperm, disappeared in epididymis while the light 40 kDa band remained present until the cauda.

For the anti-ADAM-2 N-terminal polyclonal antibody, only a very faint reactive band at about 65-70 kDa could be observed for all the epididymal sperm extracts. The anti-ADAM-2 C-terminal polyclonal antibody recognized a 90 kDa band in VE and zone 0 extracts, and a 66 kDa band from testis to the distal caput. Other faint bands at 70-75 and 50 kDa were also transiently observed in the caput while a 40 kDa appeared in zone 1 and remained until the cauda.

### Changes in ADAM-1 isoforms during maturation

**Figure 7:**
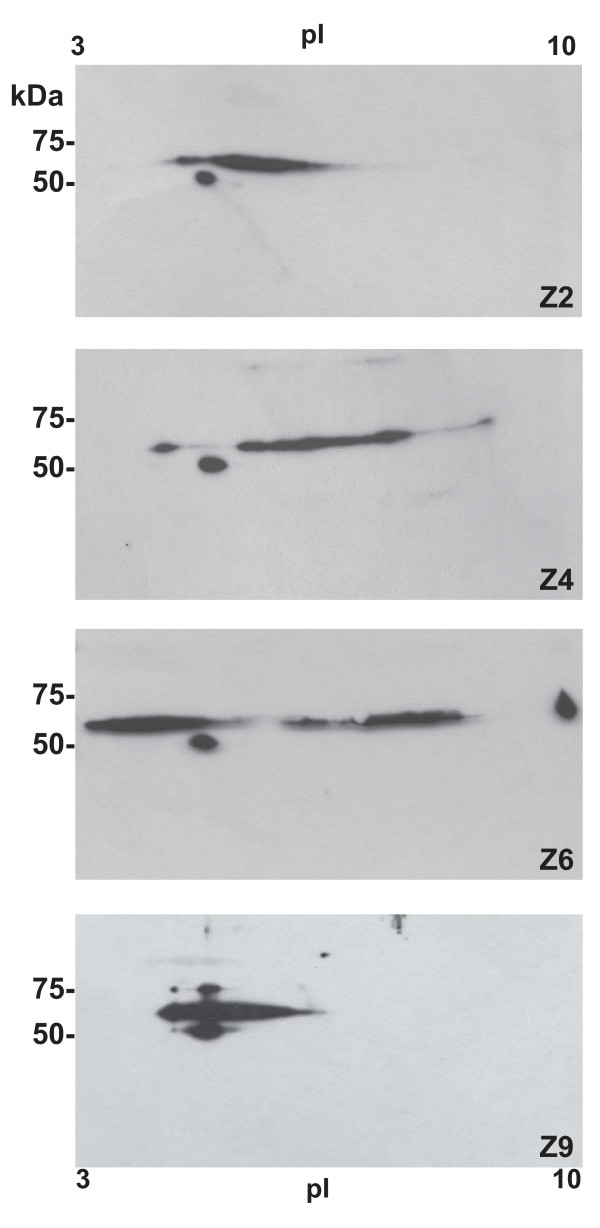
**Changes in ADAM-1 properties in bi-dimensional blots**. Sperm protein extracted from 4 different epididymal regions, proximal caput (Z2), distal caput (Z4), corpus (Z6) and distal cauda (Z9), were separated by isoelectric focusing on 8-16% SDS-PAGE and then transferred to nitrocellulose. The western blots were probed with the anti-ADAM-1 C-terminal polyclonal antibody. Several different ADAM-1 isoforms can be distinguished along the epididymis.

Detection of ADAM-1 by the anti-C-terminal polyclonal antibody provided the most intense signal on 1D gels, and this antibody was then further used on two-dimensional Western blot (Figure 7). At least three major spots were observed for ADAM-1 in the sperm extracts. The round spot at less than 50 kDa remained almost unchanged in pI and Mr in the different zones analyzed. The highly immunoreactive spot detected at about 55 kDa corresponded to at least 2 isoforms, with a smearing pattern spreading from pI 3 to 10 and a molecular mass varying between 50-55 kDa depending of the epididymal origin of the sperm extract. The less intense isoform at about 60 kDa was also present in corpus (zone 6) with a basic pI, but moved to an acidic pI in distal cauda (zone 9). In the cauda, all isoforms were found around the same acidic pI 5. Because the different isoforms were very close in masses, they were difficult to separate by one-dimensional gel electrophoresis. However, their presence likely explained the spreading of the 55 kDa band observed in immunoblots along the epididymis.

### Changes in fertilin localization on spermatozoa membrane domain during maturation

**Figure 8:**
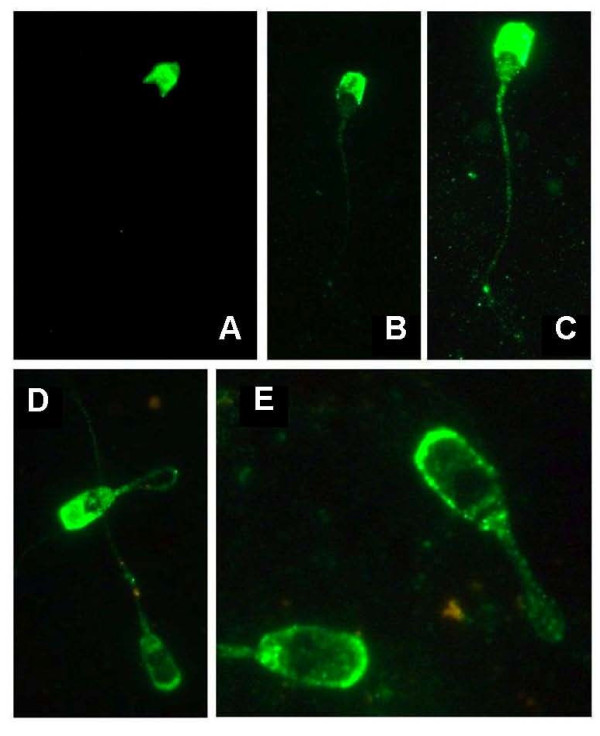
**Immunolocalization of fertilin on epididymal spermatozoa**. Sperm from caput (**A**, **B **and **C**) and cauda (**D **and **E**) epididymis were incubated with the anti-ADAM-1 C-terminal polyclonal antibody and its localization was revealed with a secondary fluorescent conjugated antibody. Sperm were from the same animal and the observations were done at × 630 (**A **and **B**) and at × 1000 (**C**, **D **and **E**).

Because the alpha-beta complex is dissociated only in presence of high SDS concentration [9], we assumed that using the ADAM-1 anti C-terminal antibody for immunolocalization studies will allow visualization of the fertilin complex on the sperm surface. Immunolocalization of fertilin complex with the anti ADAM-1-C-terminal polyclonal antibody showed that it was distributed uniformly on the acrosomal region of the sperm head throughout the caput and corpus regions, while being only present on the acrosomal ridge of spermatozoa from proximal and distal cauda (Figure 8). From caput to cauda, the sperm population with ADAM-1 on the entire acrosome declined from 94 ± 2% to 12 ± 1%, whereas the sperm population showing ADAM-1 on the acrosomal ridge increased from 6 ± 2% to 88 ± 1% (Table 1). The difference in the patterns observed between caput, corpus and cauda is statistically significant (p < 0.05).

**Table 1:** Summary of fertilin pattern distribution after immunocytochemical localization

Region	Pattern 1 Whole acrosome	Pattern2 Acrosomal ridge	Std Dev	SEM	p-value
Proximal Caput	0.9400	0.0600	0.0200	0.0115	p > 0.05^a^
Distal Caput	0.9367	0.0633	0.0058	0.0033	p > 0.05 ^a^
Proximal Corpus	0.9233	0.0767	0.0208	0.0120	p > 0.05 ^a^
Distal Corpus	0.8630	0.1370	0.0153	0.0088	p < 0.001 ^b^
Proximal Cauda	0.2100	0.7900	0.0265	0.0153	p < 0.001 ^c^
Distal Cauda	0.1200	0.8800	0.0100	0.0058	p < 0.001 ^d^

Statistical significance of the results tested by the post-hoc multiple comparison procedures (Holm-Sidak method) (p < 0.05).

### Changes in fertilin extractability during relocalization

**Figure 9:**
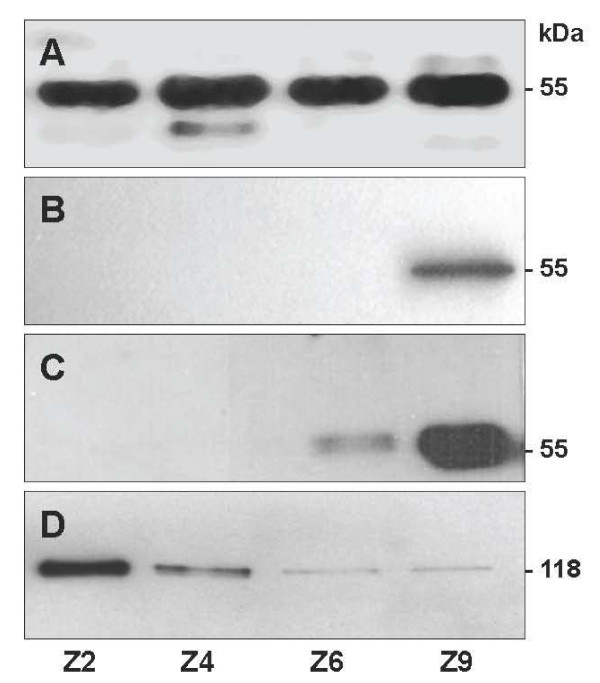
**Extractability change of ADAM-1 during sperm maturation**. Total SDS sperm protein extracts (**A**) or two different gel loading of purified biotinylated sperm surface proteins (**B **and **C**) from the proximal caput (Z2), distal caput (Z4), corpus (Z6) and distal cauda (Z9) were probed with the anti ADAM-1 C-terminal polyclonal antibody. Purified biotinylated surface proteins (**D**) were probed with the anti-ACE as controls for extraction and purification.

On total SDS sperm extracts, ADAM-1 was immunodetected as a 50 kDa band in all regions (Z2 to Z9) (Figure 9A). However, when a mild detergent treatment (NP-40) was used to extract and purified the biotin-labeled membrane surface proteins, the 50 kDa band of ADAM-1 could only be detected in cauda extracts (Figure 9B). When the gel was overloaded, a reactive band was also detected in distal corpus, but not in membrane protein extracts of either the caput or the proximal corpus (Figure 9C). When an anti-ACE was used as a control on the same sperm purified extracts, the presence of ACE was detected on all samples with the best detection for the caput sperm surface, then decreasing in the corpus and cauda, as expected for this germ cell specific enzyme that was previously described to be processed in the epididymis (Figure 9D) [17,20,21].

## Discussion

ADAM-1 and ADAM-2 are members of the metalloprotease disintegrin protein family (ADAMs) [1,22,23]. To date, at least 34 ADAMs have been identified in a variety of species and in different cells and tissues, and at least 18 of them are expressed in male reproductive organs, including the testis and epididymis [1,23]. Several of these ADAM proteins are also present on the sperm membrane, but their potential roles in fertilization are either not understood at all or need to be clarified [4-8].

In mouse, two different ADAM1 (ADAM1a and ADAM1b sharing 83% homology and 75% identity) have been previously described, that have different roles and sperm localization. ADAM1b forms a complex with ADAM2 on sperm surface, while ADAM1a is restricted to the endoplasmic reticulum of testicular germ cells [7,8]. In databases, we found two sequences for the pig ADAM-1 (that we named ADAM-1a and ADAM-1b) that shared only 76% homology. As the pig genome is still incompletely sequenced, and both sequences are located in a close position on chromosome 14 (Location 14,14 of the *Sus scrofa *reference assembly Sscrofa5), it was not possible to ascertain that these sequences indeed correspond to distinct, functional genes as shown in mouse for ADAM1a and ADAM1b. However, the PCR amplicon obtained from the pig testis (see Additional file 2, Figure S2) showed a much higher sequence identity with the pig ADAM-1a than with ADAM-1b sequence (98% vs 75%, respectively), suggesting that ADAM-1a is likely the major testicular transcript for the pig.

In boar, ADAM-1 and ADAM-2 mRNAs were present in the testis but ADAM-1 mRNA was also observed in all epididymal samples while ADAM-2 was found in the *vas efferens *and caput epididymis. In mouse, ADAM-1a mRNA is also expressed all along the mouse epididymis [24] and expression of ADAM-1 and -2 is not restricted to the male genital tract [25-27].

The process of surface fertilin maturation in epididymal spermatozoa has been mainly described in guinea pig [2,9] and mouse [8] and, recently for ADAM-2 in monkey [28]. In the present work, we show that pig ADAM-1 and ADAM-2 can be found in spermatozoa from the testis and the proximal parts of the epididymal duct under their precursor forms. However, most of ADAM-1 proteins loss the N-terminal part to be reduced to a shorter C-terminal form of about 50-55 kDa, as soon as the sperm get in the *vas efferens*. This molecular weight suggests a removal of the pro-peptide and metalloprotease domains, certainly by cleavage at the conserved pro-protein convertase site, as previously described for rodents [2]. We also observed that not all the ADAM-1 is converted, since a 70 kDa form (with the N-terminal part) is found until the distal corpus where it might be degraded or released within the sperm cytoplasmic droplet.

For ADAM-2, different bands were observed with the anti C-terminal polyclonal antibody, suggesting a sequential proteolytic cleavage pattern of maturation of boar ADAM-2: the testicular form would be processed in the proximal caput into a precursor form of 90 kDa, then to transients forms of 70-75 kDa and 50-55 kDa in the distal caput and corpus, which are further processed in the corpus leading to final form of 40-43 kDa in the cauda. This step by step processing has been reported in rodents [2,5,9], as well as suggested for other ADAM proteins of the membrane of mature spermatozoa [7,22,29-33]. ADAM-2 N-terminal antibody had a faint reactivity, only a 65-70 kDa band being observed in all sperm extracts, which corresponds to one of the transient form of ADAM-2 observed with the anti-C-terminal antibody. Different hypotheses can be proposed to explain this, including a masking of the epitope, its cleavage, or a change in its conformation once the pro-domain is removed. Anyway, these results indicate that the hydrolysis of testicular and precursors forms of ADAM-2 is a specific event restricted to proximal caput and corpus regions, respectively. In rodents, the cleavage of ADAM2 during the transit from distal corpus to proximal cauda is coincident with the relocalization of the fertilin complex on the sperm surface. In guinea pig, fertilin migrates from the whole sperm head to the post-acrosomal region, whereas in mouse it relocalizes in the equatorial region [2,34,35]. We observed a similar phenomenon in boar: in immature spermatozoa from caput and corpus, fertilin is localized over the whole acrosomal region, whereas in mature spermatozoa from cauda it is mainly concentrated on the acrosomal ridge. These results indicate that in boar the migration of fertilin occurs during sperm transit through the distal corpus and is completed during its passage through the proximal cauda. It is interesting to note that it is also in these epididymal regions that most of the membrane surface proteins and glycoproteins labeling changes have been shown to occur, [14-16] and where the number of motile and fertile spermatozoa strongly increases [36,37].

Interestingly, two-dimensional electrophoresis showed that different isoforms of the boar 50-55 kDa ADAM-1 band, differing in Mr and pI, were present in epididymal sperm extracts from the caput and the cauda. Such a variability might result from different glycosylation levels or be related to a possible cleavage of specific residues in C- or N-terminal [5,34] that would render some isoforms more hydrophobic than others and consequently make the focalization on a immobilized gradient strip more difficult. We also observed that fertilin migration or concentration to a new membrane surface domain is coincident with a different detergent extractability of at least a subset of the sperm ADAM-1. This suggests that it moves to a different lipid membrane environment, in agreement with previous data on lipid composition of the sperm membrane domains [38,39]. This change from a lipid environment to another might result from both changes in ADAM2 size and in the properties of the subset of ADAM1, due to post-translational or electrical charge modifications.

The migration of the boar fertilin to the acrosomal ridge is also in agreement with data suggesting that sperm proteins involved in primary binding with the zona pellucida are located in the apical plasma membrane of the head [38] and with a recent report indicating that fertilin beta is one of the sperm proteins retrieved on the porcine ZP [40]. Then, the relocalization in a specific spermatozoa membrane domain resulting in an increase of the local concentration of fertilin, may be crucial for sperm-oocyte interaction. We also observed two main patterns of sperm labeling in the cauda, indicating that at least two populations of spermatozoa with different maturation coexists.

In conclusion, the present work shows that in the boar the fertilin maturation involves a regionalized proteolytic processing, and the modification of both ADAM1 and ADAM-2 as well as the post-translational modifications of a subset of ADAM-1 protein. These changes may have an essential role in the migration of fertilin complexes from the whole acrosomal domain to the acrosomal ridge of the mature spermatozoa. This provides new bases to further decipher the mechanisms involved in the processing and relocalization of the sperm surface proteins during maturation, and thereof provides tools for further studies on epididymal maturation. This kind of study will be more easy in domestic mammals where testicular and epididymal spermatozoa and fluids can be obtained in larger quantity and more easily than in rodents. Moreover, the fine description of mature versus immature patterns of fertilin is an important step toward the use of this molecule as an indicator of the level of mature sperm populations in different males and toward the understanding of a potential relationship between these fertilin patterns and male fertility.

## Competing interests

The authors declare that they have no competing interests.

## Authors' contributions

AF, BG, J-LG performed the experimental work and wrote the manuscript. J-LD, MP, EP, SB participated in the design of the study and its coordination, and correct the manuscript. All authors read and approved the final manuscript.

## Supplementary Material

Additional file 1 **Figure S1**. Specificity of the polyclonal antibodies Click here for file

Additional file 2 **Figure S2**. PCR amplicons obtained from the pig testis Click here for file

Additional file 3 **Figure S3**. Absence of immunoreaction on spermatozoa with preimmune serum Click here for file
