# Supplementary material for: Expression, immunolocalization and processing of fertilins ADAM-1 and ADAM-2 in the boar (sus domesticus) spermatozoa during epididymal maturation
Source: Reprod Biol Endocrinol. 2011 Jun 30;9:96. doi: 10.1186/1477-7827-9-96 (PMC3141649; doi:10.1186/1477-7827-9-96)
Supplement: Additional file 1 — Figure S1. Specificity of the polyclonal antibodies [file 1477-7827-9-96-S1.PDF]

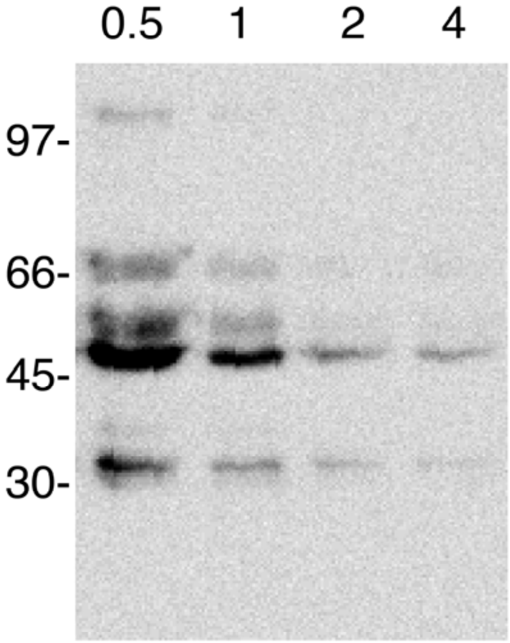

Figure 1 supplemental data. **Competition between the ADAM1 C-terminal peptide used for immunization and the ADAM1-CT antibody**.

ADAM-CT polyclonal antibody (1/10 000) was incubated overnight with increasing amount of the immunizing peptide (indicated on top in μg). When tested on a western blot from a 0.25% TX100 testis extract, a clear decrease in intensity of the reactive bands is observed indicating the specificity of the antibody. (0.5 μg of peptide has no effect on the signal as compared to the control). Although the testis sample was degraded by a long term freezing, it shows that all main reactive bands also retreived on sperm membrane (see manuscript) are competed by the peptide. (Western blot from a 12% SDS-PAGE)
