# Supplementary material for: Expression, immunolocalization and processing of fertilins ADAM-1 and ADAM-2 in the boar (sus domesticus) spermatozoa during epididymal maturation
Source: Reprod Biol Endocrinol. 2011 Jun 30;9:96. doi: 10.1186/1477-7827-9-96 (PMC3141649; doi:10.1186/1477-7827-9-96)
Supplement: Additional file 2 — Figure S2. PCR amplicons obtained from the pig testis [file 1477-7827-9-96-S2.PDF]

A : Comparison between testicular ADAM-1 amplicon sequence and pig ADAM-1a

```
Query  1     ACTGCCTCTATACTCCCTTCTCCACGGAAAAACCAAGAGCTCTTGAACGAGGCTAGCAGA  60
             |||||||||||||||||||||||||||||||||||||||||   ||||||||||||||||
Sbjct  374   ACTGCCTCTATACTCCCTTCTCCACGGAAAAACCAAGAGCTTAGGAACGAGGCTAGCAGA  433

Query  61    GGGCTTCAGACTTGGGCTCCCCAGATGAAGGGCTGGCTGCCACCCTTGCCGCCACCTTCA  120
             ||||||||||||||||||||||||||||||||||||||||||||||||||   |||||||
Sbjct  434   GGGCTTCAGACTTGGGCTCCCCAGATGAAGGGCTGGCTGCCACCCTTGCCAGGACCTTCA  493

Query  121   TGTGCCAGGTTGGCGATCATGTTGGTCTTGGTACTGATTTTCCTGCCAAGCATGTA  176
             ||||||||||||||||||||||||||||||||||||||||||||||||||||||||
Sbjct  494   TGTGCCAGGTTGGCGATCATGTTGGTCTTGGTACTGATTTTCCTGCCAAGCATGTA  549
```

Identities = 170/176 (96%) with pig ADAM-1a

B : Comparison between testicular ADAM-1 amplicon sequence and pig ADAM-1b

```
Query  1     ACTGCCTCTATACTCCCTTCTCCACGGAAAAACCAAGAGCTCTTGAACGAGGCTAGCAGA  60
             |||||||||||||||||||||||||||||||| |||| |  ||||||  ||| ||  | |
Sbjct  319   ACTGCCTCTATACTCCCTTCTCCACGGAAAAATCAAGTGGCCTTGAAAAAGGGTAAGATA  378

Query  61    GGGCTTCAGACTTGGGCTCCCCAGATGAAGGGCT---GGCTGCCACCCTTGCCGCCACCT  117
               | |||||||||||||||  |||| ||||| ||   || ||   ||  ||||   | ||
Sbjct  379   AAGTTTCAGACTTGGGCTCAGCAGAAGAAGGACTTGAGGTTGAGGCCAGTGCCAGGATCT  438

Query  118   TCATGTGCCAGGTTGGCGATCATGTTGGTCTTGGTACTGATTTTCCTGCCAAGCATGTA  176
             ||||||  ||| |||  ||   | ||  | ||||   |||||||||||||||||||||
Sbjct  439   TCATGTATCAGTTTGAGGACTGTCTTTCTGTTGG---TGATTTTCCTGCCAAGCATGTA  494
```

Identities = 134/179 (74%) with pig ADAM-1b

C : Comparison between testicular ADAM-2 amplicon sequence and pig ADAM-2

```
Query  6      ATGTGACTGTGGGACCCCGGAGAATTGTGCTGCTCAGCCAAACGCATGCTGTAACCAGGC  65
              ||||||||||||||||||||||||||||||||||||||||||||||||||||||||||||
Sbjct  1285   ATGTGACTGTGGGACCCCGGAGAATTGTGCTGCTCAGCCAAACGCATGCTGTAACCAGGC  1344

Query  66     CACCTGTACACTCTCAGCTGGCTCTGCCTGTGCCACTGGGCCGTGCTGTGATTCCTGTTC  125
              ||||||||||||||||||||||||||||||||||||||||||||||||||||||||||||
Sbjct  1345   CACCTGTACACTCTCAGCTGGCTCTGCCTGTGCCACTGGGCCGTGCTGTGATTCCTGTTC  1404

Query  126    TTTTATGGCAAAAGGACAAACATGTAGGCTTACCTTGGATGAATGCGATCTCCTTGAATA  185
              ||||||||||||||||||||||||||||||||||||||||||||||||||||||||||||
Sbjct  1405   TTTTATGGCAAAAGGACAAACATGTAGGCTTACCTTGGATGAATGCGATCTCCTTGAATA  1464

Query  186    TTGCAATGGTTCTTCTGCAGCATGTCA  212
              |||||||||||||||||||||||||||
Sbjct  1465   TTGCAATGGTTCTTCTGCAGCATGTCA  1491
```

Identities = 207/207 (100%) with Sus scrofa mRNA for fertilin beta (FTNB gene)
