# Supplementary material for: Expression, immunolocalization and processing of fertilins ADAM-1 and ADAM-2 in the boar (sus domesticus) spermatozoa during epididymal maturation
Source: Reprod Biol Endocrinol. 2011 Jun 30;9:96. doi: 10.1186/1477-7827-9-96 (PMC3141649; doi:10.1186/1477-7827-9-96)
Supplement: Additional file 3 — Figure S3. Absence of immunoreaction on spermatozoa with preimmune serum [file 1477-7827-9-96-S3.PDF]

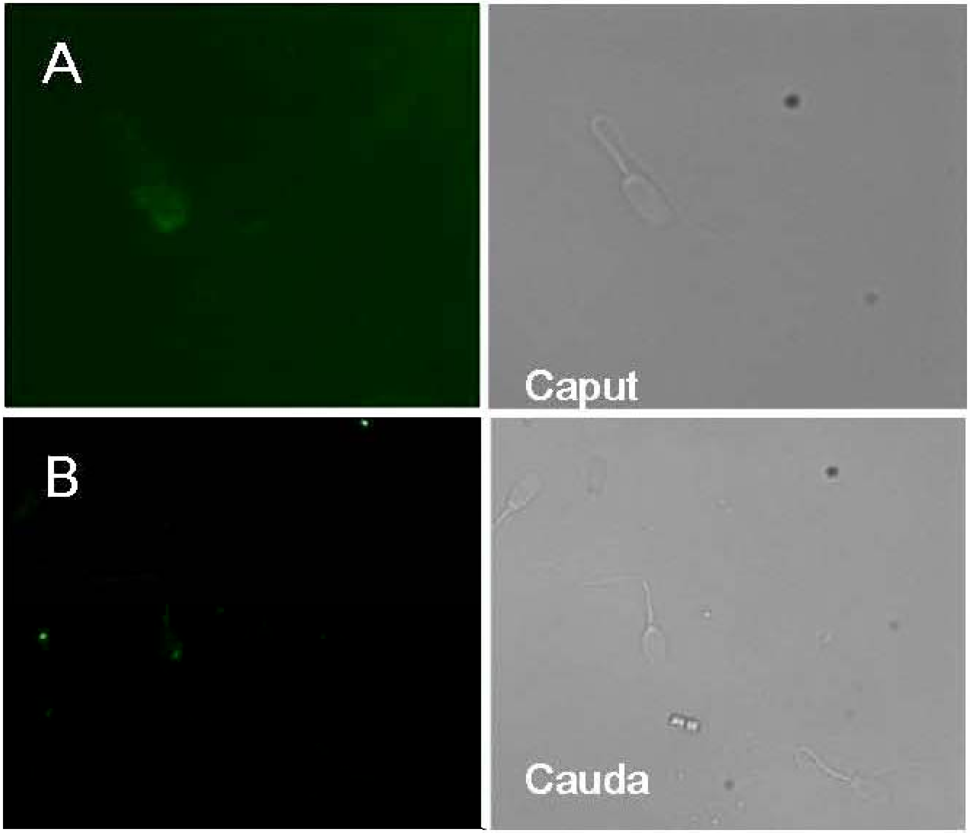

Supplemented data, Figure 3 . Immunolocalization with the preimmune fertilin antibody on epididymal sperm (**A**) from caput and (**B**) from cauda. Spermatozoa were incubated with the preimmune polyclonal serum as described in the materials and methods and revealed with a second anti-rabbit alexa fluor 488 conjugated fluorescent antibody. Sperm were from the same animal and the observations were done at x 630 magnifications. Only a fuzzy low intensity labeling could be observed.
